# Gene expression programs during callus development in tissue culture of two Eucalyptus species

**DOI:** 10.1186/s12870-021-03391-x

**Published:** 2022-01-03

**Authors:** Ye Zhang, Junji Li, Changrong Li, Shengkan Chen, Qinglan Tang, Yufei Xiao, Lianxiang Zhong, Yingying Chen, Bowen Chen

**Affiliations:** Guangxi Key Laboratory of Superior Timber Trees Resource Cultivation, Guangxi Forestry Research Institute, 23 Yongwu Road, Nanning, 530002 Guangxi China

**Keywords:** Eucalyptus, callus, vegetative propagation, somatic embryogenesis, transcriptome, transcription factor

## Abstract

**Background:**

Eucalyptus is a highly diverse genus of the *Myrtaceae* family and widely planted in the world for timber and pulp production. Tissue culture induced callus has become a common tool for Eucalyptus breeding, however, our knowledge about the genes related to the callus maturation and shoot regeneration is still poor.

**Results:**

We set up an experiment to monitor the callus induction and callus development of two Eucalyptus species - *E. camaldulensis* (high embryogenic potential) and *E. grandis x urophylla* (low embryogenic potential). Then, we performed transcriptome sequencing for primary callus, mature callus, shoot regeneration stage callus and senescence callus. We identified 707 upregulated and 694 downregulated genes during the maturation process of the two Eucalyptus species and most of them were involved in the signaling pathways like plant hormone and MAPK. Next, we identified 135 and 142 genes that might play important roles during the callus development of *E. camaldulensis* and *E. grandis x urophylla*, respectively. Further, we found 15 DEGs shared by these two Eucalyptus species during the callus development, including Eucgr.D00640 (stem-specific protein TSJT1), Eucgr.B00171 (BTB/POZ and TAZ domain-containing protein 1), Eucgr.C00948 (zinc finger CCCH domain-containing protein 20), Eucgr.K01667 (stomatal closure-related actinbinding protein 3), Eucgr.C00663 (glutaredoxin-C10) and Eucgr.C00419 (UPF0481 protein At3g47200). Interestingly, the expression patterns of these genes displayed “N” shape in the samples. Further, we found 51 genes that were dysregulated during the callus development of *E. camaldulensis* but without changes in *E. grandis x urophylla*, such as Eucgr.B02127 (GRF1-interacting factor 1), Eucgr.C00947 (transcription factor MYB36), Eucgr.B02752 (laccase-7), Eucgr.B03985 (transcription factor MYB108), Eucgr.D00536 (GDSL esterase/lipase At5g45920) and Eucgr.B02347 (scarecrow-like protein 34). These 51 genes might be associated with the high propagation ability of Eucalyptus and 22 might be induced after the dedifferentiation. Last, we performed WGCNA to identify the co-expressed genes during the callus development of Eucalyptus and qRT-PCR experiment to validate the gene expression patterns.

**Conclusions:**

This is the first time to globally study the gene profiles during the callus development of Eucalyptus. The results will improve our understanding of gene regulation and molecular mechanisms in the callus maturation and shoot regeneration.

**Supplementary Information:**

The online version contains supplementary material available at 10.1186/s12870-021-03391-x.

## Background

The regeneration of plant tissue or organs under culture conditions has been extensively used for decades in plants. Plants generate callus in response to stresses like wounding and pathogen infection, and callus cells are totipotent and are able to regenerate the whole plant body [1]. Depending on the organs they generate, calli with some degrees of organ regeneration are called with different names, such as rooty, shooty, embryogenic callus, and compact callus [2, 3]. *In vitro*, exogenous application of auxin and cytokine has been proved to induce callus in plants. For example, Skoog and Miller showed that a high ratio of auxin-to-cytokinin can induce root regeneration, while a high ratio of cytokinin-to-auxin can induce shoot regeneration [4]. In nature, wounding, pathogens and interspecific hybrids are common ways to induce callus and tumors [1]. Some molecules have been identified to play key roles during the callus induction and development. For example, lateral organ boundaries domain (LBD) family of transcription factors (e.g., LBD16, LBD17, LBD18, LBD29) can mediate the expression of auxin response factors ARF7 and ARF19 [5, 6]. ARR1 and ARR21 have been identified to induce callus formation in *Arabidopsis* [7, 8]. RWP-RK domain transcription factors like RKD1, RKD2 and RKD4 have been found to mediate the gametogenesis and embryogenesis [9, 10]. In addition, some studies have been demonstrated to uncover the genes and proteins involved in the callus development. Tan et al. identified 73 proteins significantly differentially expressed during the callus development in *Vanilla planifolia* Andrews [11]. Che et al. identified RAP2.6L as a key factor for shoot regeneration in Arabidopsis because the T-DNA knockdown mutations in RAP2.6L reduced the expression of many genes that are normally up-regulated during shoot development [12]. However, our knowledge about the genes involved in the callus development and tissue regeneration process in plants is still poor.

Eucalyptus, a highly diverse genus of the *Myrtaceae* family, is widely planted in the world due to its significant economic values for timber and pulp [13]. As we know, all commonly recognized methods of vegetative propagation have been applied with Eucalyptus, but most have resulted in failure especially when applied to adult tissues [13]. Successful regeneration of plants or organs from selected Eucalyptus has never been reported until 1981 when callus was induced on embryos and sterile seedlings of selected trees of *E. leichow* [13]. Then, this technique was applied with many Eucalyptus species like *E. polybractea*, *E. cama1dulensis*, *E. gomphocephala* and *E. viminalis* [13]. Another technique that has been successfully applied with Eucalyptus is organ culture, in which differentiated tissues such as leaves, stems and roots are placed in a controlled system of nutrients and environment. Roots and/or buds can be induced on the explant either directly or after the formation of a callus and many tree species, including Eucalyptus, which can be propagated by organ culture techniques have applied with this method. Although organ culture techniques are often used in preference to the traditional methods of vegetative propagation due to its high multiplication rates, there are some problems usually happened in developing the organ culture techniques, such as obtaining aseptic tissue from field-grown plants, brown exudate, rooting and bud inhibitors [13]. Also, some factors have been reported to affect the root initiation in nodes of Eucalyptus trees, such as the culture medium, the incubation conditions, the physiological state of the parent plant, and the position on the parent plan t[1, 14].

Some vegetative propagation associated studies have been demonstrated in Eucalyptus. Grattapaglia et al. identified some QTLs controlling the ability to form shoots of *E. grandi s*[15]. Marques et al. identified QTLs related to adventitious rooting, sprouting ability and the stability of adventitious rooting [16]. In plants, some genes have been reported to play key roles during the vegetative propagation, such as ARF19, SERK, LEC and WUS [17, 18]. Previously, our lab reported the transcriptome profiles of two Eucalyptus species during somatic embryogenesis and dedifferentiation [19]. We identified genes encoding somatic embryogenesis receptor kinase, ethylene, auxin, ribosomal protein, zinc finger protein, heat shock protein, histone, cell wall related protein and multiple transcription factors that might control the ability of somatic embryogenesis and dedifferentiation. However, large is unknown about the gene regulations during the developmental process after the callus is induced in Eucalyptus.

In the present study, we aimed to investigate the transcriptome profiles of dedifferentiated callus tissues incubated on the culture medium. We also aimed to identify genes involved in the callus development process and controlling the ability of vegetative propagation. This is the first time to study the gene profiles of dedifferentiated callus tissues of Eucalyptus and our results will provide new insights of understanding the molecular mechanisms in the callus development and differentiation processes. More importantly, our results will improve our knowledge about the genes associated with the vegetative propagation ability of Eucalyptus.

## Results

### Callus induction and incubation

**Fig. 1:**
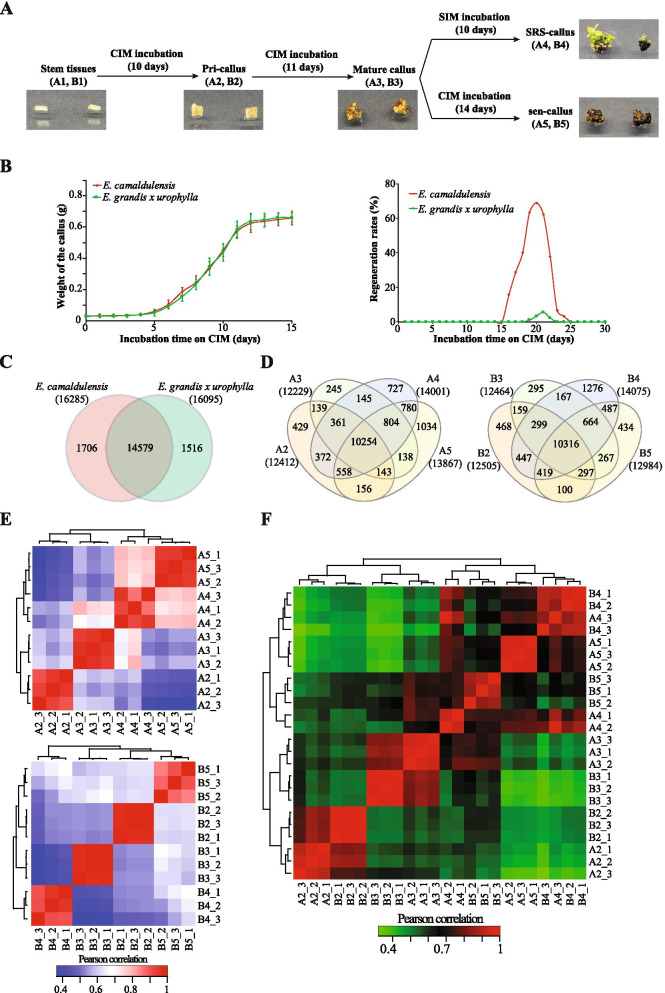
Callus induction, development, and transcriptome sequencing. (A) Experimental design of the callus induction and development. Stem tissues (A1, B1) were incubated on the CIM for dedifferentiation to get primary callus (A2, B2, pri-callus), which were further incubated on CIM for maturation (A3, B3, mat-callus). Mature calluses were transferred onto the SIM for 10 days incubation to expand the buds (A4, B4), as the shoot regeneration stage callus (SRS-callus). While mature calluses incubated on the CIM for long time (14 days) would lose the regeneration ability, which were called senescence callus (A5, B5, sen-callus). (B) Physiological experiments of callus during the incubation. Left and right panels are the weight and regeneration rates of the callus incubated on the medium for different time lengths. (C) Venn diagram of genes identified in the two Eucalyptus species. (D) Venn diagrams of genes identified in the callus tissues during the development of *E. camaldulensis* (left) and *E. grandis x urophylla* (right). (E) Correlation heat maps of the samples based on the gene expression profiles of callus tissues during the development of the two Eucalyptus species. (F) Combined correlation heat map of all the samples used in this study

To understand the gene expression profiles during the callus development of Eucalyptus, we obtained the stem tissues of two Eucalyptus species – *E. camaldulensis* (high embryogenic potential, A1) and *E. grandis x urophylla* (low embryogenic potential, B1). We performed the *in vitro* tissue-culture experiments on these stem samples and obtained callus tissues from different developmental stages (Figure 1A). Initially, we observed that the incubation time on CIM (callus-inducing medium) of the stems has a great impact on their regeneration ability. As shown in Figure 1B, the regeneration rates of the tissue culture induced callus by stem peaked at 21 days of incubation on CIM. It is notable that the regeneration rate of *E. camaldulensis* callus was much higher than *E. grandis x urophylla*. We obtained the callus (also called primary callus, pri-callus) at 10 days (A2 and B2) and mature callus at 21 days (A3 and B3). Then, the mature callus tissues were transferred to SIM (shooting-inducing medium) for further incubation. They were shown to start generating buds after 7 days incubation and 80% of the callus generated buds after 10 days incubation. We obtained the tissues of callus tissues incubated on SIM for 10 days for the two Eucalyptus species (A4 and B4). It is interesting that the callus tissues turned brown intensively, the bead-like protrusions also turned brown to black, and the callus lost the regeneration ability after incubation on CIM for 30 days. The callus tissues incubated on CIM for 35 days were obtained for the two species of Eucalyptus (A5 and B5).

### Transcriptome sequencing and gene expression profiles

We employed the transcriptome sequencing for the callus tissues of *E. camaldulensis* (A2~A5) and *E. grandis x urophylla* (B2~B5). After data cleaning, we obtained 20.99 to 23.58 million reads for these samples and found 69.27% to 84.58% of the clean reads mapped to the reference Eucalyptus genome. Next, we used StringTie to identify genes expressed in the callus tissues of the two Eucalyptus species. After the average TPM (transcripts per million reads) values of all genes were calculated and lowly expressed genes (TPM < 5) were filtered, we identified 12,229 to 14,075 genes for all the samples. It showed in Figure 1C that 14,579 genes were identified in the callus tissues of both *E. camaldulensis* and *E. grandis x urophylla* and that 1,706 and 1,516 genes were expressed specifically in *E. camaldulensis* and *E. grandis x urophylla*, respectively. Then, we compared the genes identified in different stages of callus tissues. Figure 1D showed that 10,254 and 10,316 genes were commonly identified in all the callus tissues of *E. camaldulensis* and *E. grandis x urophylla*, respectively. Next, we analyzed the sample correlation during the callus development using the gene expression profiles. As expected, the replicates were performed well, and the samples can be distinguished from each other based on the gene expression profiles (Figure 1E). Further, we analyzed the gene expression profiles across species. Notably, the callus tissues showed similarities between *E. camaldulensis* and *E. grandis x urophylla* before mature callus developmental stage (Figure 1F), which indicates that the callus differentiation process varies in these two Eucalyptus species on molecular level. Based on the developmental stages, we divided the whole process into three parts to investigate the gene changes during the differentiation process, including pri-callus to mature callus (mat-callus), mature callus to shoot regeneration stage callus (SRS-callus), and mature callus to senescence callus (sen-callus).

### DEGs in callus maturation

**Fig. 2:**
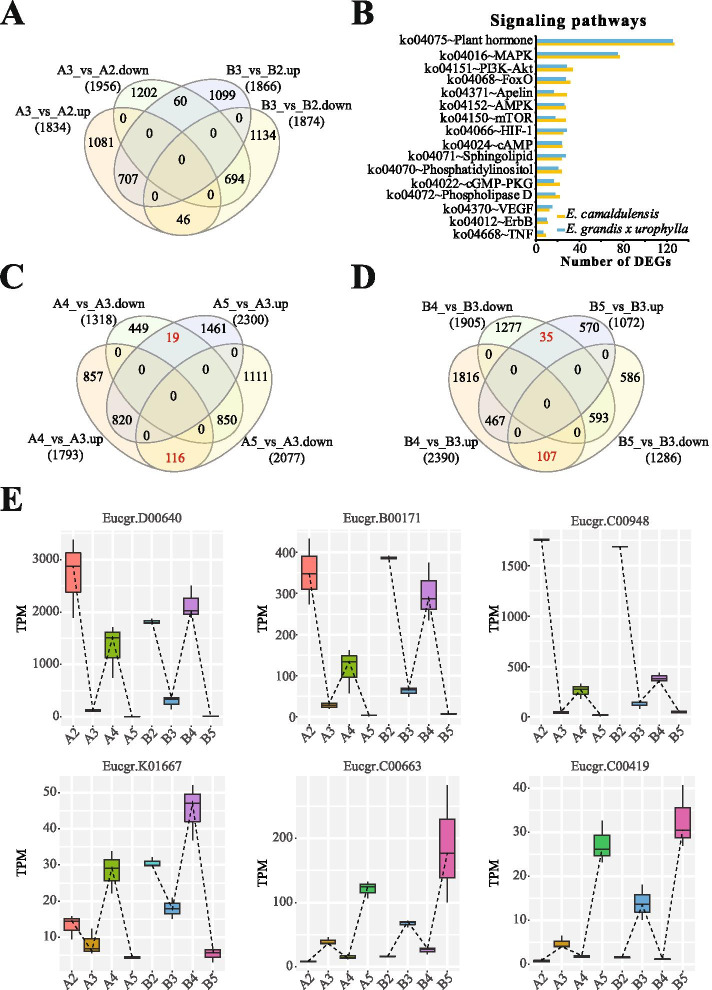
Differential expression analysis identified genes associated with the callus maturation and development in the two Eucalyptus species. (A) Venn diagram of DEGs identified in the maturation process of the two Eucalyptus species. (B) Signalling pathways involved by the DEGs during the callus maturation in Eucalyptus. (C) DEGs identified in the SRS-callus and sen-callus compared with mat-callus of *E. camaldulensis*. Numbers in red represent the genes with diverse regulations in SRS-callus and sen-callus compared with mature callus. (D) DEGs identified in the SRS-callus and sen-callus compared with mature callus of *E. grandis x urophylla*. Numbers in red represent the genes with diverse regulations in SRS-callus and sen-callus compared with mature callus. (E) Box plots showing the expression patterns of six genes in the callus development of *E. camaldulensis*

We compared the gene expression profiles of primary and mature callus tissues in the two Eucalyptus species. Initially, we identified 3,790 (1,834 upregulated and 1,956 downregulated) and 3,740 (1,834 upregulated and 1,956 downregulated) DEGs in *E. camaldulensis* (A3 compared to A2) and *E. grandis x urophylla* (B3 compared to B2), respectively (Figure 2A, additional file 1). In this process, the two Eucalyptus species shared 707 upregulated and 694 downregulated genes (Figure 2A). Notably, 106 genes were found with adverse regulation during the callus maturation process in the two Eucalyptus species (Figure 2A), including Eucgr.I01667 (kelch repeat-containing protein At3g27220), Eucgr.B00093 (HVA22-like protein e), Eucgr.G02764 (glutathione S-transferase DHAR2), Eucgr.F00590 (snakin-2), Eucgr.D00272 (CBL-interacting serine/threonine-protein kinase 23) and Eucgr.F00184 (low-temperature-induced cysteine proteinase). We next analyzed the signal transduction pathways involved by the DEGs. Notably, 127 and 125 DEGs were enriched in the plant hormone signaling transduction pathway of *E. camaldulensis* and *E. grandis x urophylla*, respectively (Figure 2B). In addition, we found 77 and 75 DEGs enriched in the plant MAPK signaling pathway of *E. camaldulensis* and *E. grandis x urophylla*, respectively (Figure 2B). Gene Ontology enrichment analysis identified that 165 and 136 DEGs were involved in the protein phosphorylation (GO:0006468) during the callus maturation process of *E. camaldulensis* and *E. grandis x urophylla*, respectively. Further, we compared the dysregulated genes during the process of stem to mature callus. It showed in Table 1 that 40 upregulated and 34 downregulated genes were identified in this process, and they might be involved in the dedifferentiation and callus development.

**Table 1:** Genes involved in the callus maturation of the two Eucalyptus species.

Regulation	GeneID	*E. camaldulensis*				*E. grandis x urophylla*				Description
		A2 vs A1		A3 vs A2		B2 vs B1		B3 vs B2		
		Log2FC	FDR	Log2FC	FDR	Log2FC	FDR	Log2FC	FDR	
UP	Eucgr.A01725	3.988	5.79E-19	1.240	4.09E-03	3.050	1.92E-11	3.235	9.39E-14	uncharacterized LOC104439796
	Eucgr.A02688	1.118	1.03E-02	4.595	4.46E-24	3.856	1.00E-11	3.567	1.20E-15	ABC transporter G family member 4
	Eucgr.B00048	1.199	4.53E-03	3.946	2.57E-19	1.793	3.74E-05	3.632	1.36E-16	transcription factor bHLH96
	Eucgr.B00168	2.934	8.60E-12	2.715	1.30E-10	2.714	2.42E-10	1.202	6.13E-03	probable pectate lyase 18
	Eucgr.B01206	4.818	2.46E-14	1.551	4.94E-04	1.238	1.58E-02	2.670	1.47E-09	
	Eucgr.B01809	4.392	1.54E-22	1.529	3.07E-04	3.187	1.45E-13	2.768	7.51E-11	pleiotropic drug resistance protein 3
	Eucgr.B02181	8.123	1.57E-32	1.294	3.02E-03	3.175	1.94E-09	2.840	1.01E-10	
	Eucgr.B02604	4.143	3.25E-20	1.232	4.31E-03	1.838	1.49E-05	1.038	2.03E-02	G-type lectin S-receptor-like serine/threonine-protein kinase LECRK2
	Eucgr.B03352	1.972	1.94E-06	1.367	1.40E-03	1.994	1.75E-06	1.894	7.76E-06	uncharacterized LOC104433550
	Eucgr.C00355	1.182	4.68E-03	1.645	1.00E-04	1.023	1.52E-02	1.342	1.89E-03	reticulon-like protein B21
	Eucgr.C02352	2.709	3.60E-10	1.818	1.69E-05	2.520	4.41E-09	1.295	3.00E-03	serine carboxypeptidase-like 18
	Eucgr.D00605	1.414	6.85E-04	1.073	1.48E-02	1.275	2.40E-03	1.094	1.35E-02	putative leucine-rich repeat receptor-like serine/threonine-protein kinase At2g14440
	Eucgr.D00954	4.836	1.42E-22	1.073	1.55E-02	2.575	9.42E-09	1.254	4.75E-03	E3 ubiquitin-protein ligase RGLG2
	Eucgr.D01675	2.538	2.28E-08	1.936	5.81E-06	3.383	1.39E-11	2.348	5.29E-08	endoglucanase 1
	Eucgr.D01945	2.412	6.81E-09	4.119	1.13E-20	1.866	6.50E-06	1.246	4.15E-03	leucoanthocyanidin dioxygenase
	Eucgr.E00448	1.234	2.77E-03	4.526	7.15E-24	2.524	1.27E-09	1.509	4.14E-04	14 kDa proline-rich protein DC2.15
	Eucgr.E01134	2.541	9.67E-10	3.023	1.09E-12	2.593	5.39E-10	1.902	6.82E-06	probable methyltransferase PMT18
	Eucgr.E01615	7.981	6.65E-14	1.577	5.86E-04	4.518	7.78E-12	2.426	4.86E-08	putative expansin-B2
	Eucgr.E03269	8.688	1.92E-18	9.505	3.15E-64	8.012	8.70E-26	10.092	4.41E-70	non-classical arabinogalactan protein 31
	Eucgr.E04292	5.690	5.43E-24	1.459	7.59E-04	2.991	3.33E-11	1.212	6.27E-03	UPF0481 protein At3g47200
	Eucgr.F00411	3.742	4.57E-18	3.997	9.18E-20	2.626	2.96E-10	2.174	2.47E-07	aspartyl protease AED3
	Eucgr.F02401	3.559	3.00E-08	2.716	1.46E-09	6.099	2.23E-24	1.141	1.13E-02	probable transcription factor KAN4
	Eucgr.G00401	1.091	1.28E-02	1.284	3.45E-03	1.209	6.03E-03	1.169	9.09E-03	cleavage and polyadenylation specificity factor subunit 3-I
	Eucgr.G01473	6.830	6.75E-29	1.610	1.81E-04	2.197	2.83E-06	3.177	4.69E-13	
	Eucgr.G02798	3.384	7.51E-13	5.107	4.05E-28	4.317	1.04E-20	1.860	1.16E-05	probable RNA-binding protein ARP1
	Eucgr.H01056	1.115	1.42E-02	4.519	2.69E-23	1.297	2.51E-03	2.876	1.97E-11	hypothetical protein
	Eucgr.H03413	9.211	3.08E-35	1.313	2.49E-03	6.335	3.58E-26	2.820	5.35E-11	1,2-dihydroxy-3-keto-5-methylthiopentene dioxygenase 2
	Eucgr.I01142	2.106	4.59E-05	2.475	2.22E-08	2.985	1.31E-08	1.792	5.61E-05	ferric reduction oxidase 6
	Eucgr.I01304	2.870	1.01E-09	1.467	8.33E-04	1.050	1.72E-02	3.921	1.15E-18	purple acid phosphatase 2
	Eucgr.I01381	2.654	8.51E-10	1.958	3.64E-06	2.552	2.66E-09	2.122	5.36E-07	
	Eucgr.I01777	4.087	5.93E-12	1.885	2.00E-05	4.192	1.81E-14	2.759	2.06E-10	polygalacturonase At1g48100
	Eucgr.I02745	3.632	2.78E-14	3.328	1.72E-14	6.260	4.49E-29	2.229	1.57E-07	MLO-like protein 4
	Eucgr.J00003	4.833	9.79E-17	1.648	1.76E-04	2.818	2.97E-10	1.612	1.87E-04	elicitor-responsive protein 1
	Eucgr.J01192	3.766	2.71E-15	1.009	2.51E-02	3.607	6.08E-16	1.497	4.96E-04	annexin-like protein RJ4
	Eucgr.J02174	2.412	8.40E-06	5.483	3.86E-30	5.373	3.49E-12	6.790	1.94E-40	gibberellin-regulated protein 14
	Eucgr.K00156	2.080	4.90E-07	2.024	1.39E-06	1.860	7.07E-06	1.666	9.11E-05	monocopper oxidase-like protein SKS1
	Eucgr.K00648	1.123	8.69E-03	2.478	4.91E-09	1.470	6.18E-04	1.353	1.95E-03	glucan endo-1,3-beta-glucosidase 12
	Eucgr.K00883	2.378	8.22E-09	2.150	2.80E-07	1.971	1.73E-06	1.248	4.04E-03	xyloglucan endotransglucosylase/hydrolase protein 9
	Eucgr.K02477	4.609	3.28E-12	2.164	1.09E-06	1.756	3.86E-05	1.437	9.42E-04	GDSL esterase/lipase At1g74460
	Eucgr.L01603	2.172	3.18E-06	2.297	9.75E-08	1.105	8.83E-03	1.610	1.64E-04	
DOWN	Eucgr.A00020	-1.542	1.65E-04	-1.372	1.31E-03	-2.412	6.05E-09	-1.367	1.56E-03	endochitinase EP3
	Eucgr.A00514	-2.893	4.82E-12	-2.556	1.69E-09	-2.640	2.54E-10	-2.627	8.95E-10	auxin transporter-like protein 4
	Eucgr.A02039	-1.070	1.13E-02	-4.313	5.60E-19	-1.923	3.88E-06	-4.530	7.41E-20	oxygen-evolving enhancer protein 2, chloroplastic
	Eucgr.A02983	-2.811	4.26E-11	-4.482	4.11E-18	-3.107	7.08E-13	-2.144	5.32E-06	S-norcoclaurine synthase 1
	Eucgr.B01742	-1.183	4.83E-03	-5.711	1.80E-25	-3.181	7.03E-14	-5.841	1.40E-28	
	Eucgr.B02326	-1.134	7.07E-03	-1.007	2.64E-02	-1.187	4.68E-03	-2.798	1.95E-10	uncharacterized LOC104432710
	Eucgr.B03947	-3.575	1.81E-16	-4.742	2.32E-19	-2.842	1.92E-11	-2.254	4.06E-07	uncharacterized LOC104434068
	Eucgr.C01002	-2.170	2.53E-07	-1.125	1.57E-02	-2.971	2.93E-12	-1.079	1.95E-02	2-oxoglutarate-Fe(II) type oxidoreductase
	Eucgr.C03807	-2.058	5.72E-07	-2.397	2.15E-08	-2.390	9.02E-09	-1.065	1.78E-02	transcription factor bHLH68
	Eucgr.D00409	-2.792	3.24E-11	-1.072	1.77E-02	-2.019	1.00E-06	-2.048	1.40E-06	GDSL esterase/lipase At5g45670
	Eucgr.D02537	-1.085	9.49E-03	-4.459	1.48E-20	-1.048	1.29E-02	-5.198	4.10E-26	actin-7
	Eucgr.E03614	-2.539	9.66E-10	-2.610	1.25E-09	-4.023	4.19E-20	-2.912	2.03E-11	transcription factor bHLH112
	Eucgr.F00225	-1.233	2.98E-03	-1.353	1.93E-03	-1.233	3.44E-03	-1.447	1.10E-03	abscisic acid 8'-hydroxylase 4
	Eucgr.F00746	-4.799	2.42E-26	-3.242	1.00E-13	-6.510	4.04E-40	-3.012	2.59E-11	GDSL esterase/lipase At5g55050
	Eucgr.F00751	-1.512	6.99E-04	-2.021	9.46E-05	-2.998	5.20E-12	-1.833	1.99E-04	CASP-like protein 2D1
	Eucgr.F01151	-4.291	2.67E-22	-1.389	1.19E-03	-4.169	3.06E-21	-3.681	1.17E-16	early nodulin-like protein 2
	Eucgr.F03054	-1.561	1.39E-04	-1.731	4.18E-05	-2.068	5.47E-07	-1.117	1.15E-02	aquaporin TIP2-1
	Eucgr.F03476	-1.384	8.19E-04	-2.657	7.78E-10	-3.019	1.72E-12	-1.462	1.25E-03	
	Eucgr.F03502	-1.444	7.97E-04	-2.955	1.25E-09	-1.379	1.16E-03	-1.633	3.13E-04	mitochondrial pyruvate carrier 4
	Eucgr.F04099	-2.419	1.38E-08	-2.958	1.08E-09	-2.032	1.23E-06	-5.325	3.00E-22	chlorophyll a-b binding protein CP26, chloroplastic
	Eucgr.G00777	-1.489	4.68E-04	-3.508	1.58E-13	-2.124	9.03E-07	-3.395	1.96E-11	chlorophyll a-b binding protein CP24 10A, chloroplastic
	Eucgr.G03066	-2.205	9.40E-08	-4.927	2.06E-23	-4.869	1.78E-26	-2.162	1.36E-06	leucoanthocyanidin dioxygenase
	Eucgr.H00595	-3.934	2.39E-19	-1.694	9.95E-05	-2.535	1.14E-09	-1.268	3.73E-03	glutamate decarboxylase
	Eucgr.H02266	-1.601	1.11E-04	-2.894	6.48E-11	-1.154	6.56E-03	-2.809	4.85E-10	metal transporter Nramp3
	Eucgr.H03958	-2.417	7.88E-09	-4.511	4.43E-19	-1.978	2.65E-06	-2.671	1.10E-08	bifunctional epoxide hydrolase 2
	Eucgr.H03987	-2.562	3.69E-08	-2.992	9.99E-08	-4.309	5.54E-21	-1.351	9.37E-03	major allergen Pru ar 1
	Eucgr.H03989	-4.077	7.12E-20	-1.402	2.79E-03	-3.295	2.23E-14	-1.011	3.21E-02	major allergen Pru ar 1
	Eucgr.I01368	-1.958	2.23E-06	-3.345	1.54E-13	-1.115	7.84E-03	-1.826	2.14E-05	uncharacterized LOC104418905
	Eucgr.I02500	-1.980	2.51E-06	-1.815	6.17E-05	-1.273	2.43E-03	-1.108	1.44E-02	uncharacterized LOC104419798
	Eucgr.J00422	-1.710	3.38E-05	-4.162	8.47E-19	-1.096	8.92E-03	-3.003	4.21E-12	uncharacterized protein At4g22758
	Eucgr.J01502	-2.483	2.02E-09	-4.570	3.84E-23	-3.523	2.33E-16	-4.177	4.47E-20	ribulose bisphosphate carboxylase small chain, chloroplastic
	Eucgr.K01283	-1.963	1.71E-06	-3.971	8.59E-19	-1.160	5.38E-03	-2.618	7.12E-10	non-specific lipid-transfer protein 1
	Eucgr.K01691	-2.122	2.59E-07	-2.412	2.13E-08	-1.829	9.90E-06	-2.053	2.00E-06	alpha-L-arabinofuranosidase 1
	Eucgr.K03296	-1.119	7.26E-03	-4.504	8.36E-22	-1.971	2.14E-06	-2.787	2.60E-10	cytochrome P450 94A1

### DEGs in callus development

We next identified DEGs in the differentiation process of callus in the two Eucalyptus species. Compared to A3, we identified 3,111 (1,793 upregulated and 1,318 downregulated) and 4,377 (2,300 upregulated and 2,077 downregulated) genes differentially expressed in A4 and A5, respectively (Figure 2C, additional file 1). As A4 has the propagation ability while A5 lost it, we next investigated the genes with diverse regulations in A4 and A5. It showed that a total of 135 genes with such characteristics (additional file 2). These genes were predicted to be enriched in the biological processes like “GO:0048827~phyllome development” (1 gene, Eucgr.B02127), “GO:0055114~oxidation-reduction process” (14 genes) and “GO:0009791~post-embryonic development” (1 gene, Eucgr.I02367). Likewise, compared to B3 we identified 4,295 (2,390 upregulated and 1,905 downregulated) and 2,358 (1,072 upregulated and 1,286 downregulated) genes differentially expressed in B4 and B5, respectively (Figure 2D). There were 142 genes with diverse regulations in B4 and B5 (additional file 2). These genes were enriched in the biological processes including “GO:0006542~glutamine biosynthetic process” (1 gene, Eucgr.G02570), “GO:0031145~anaphase-promoting complex-dependent catabolic process” (1 gene, Eucgr.J00733), “GO:0044765~single-organism transport” (1 gene, Eucgr.A00992), “GO:0006952~defense response” (1 gene, Eucgr.F03332), “GO:0016569~covalent chromatin modification” (1 gene, Eucgr.J03029), “GO:0008643~carbohydrate transport” (1 gene, Eucgr.C02790) and “GO:0006012~galactose metabolic process” (1 gene, Eucgr.C02197).

Further, we compared the DEGs with diverse regulations in SRS-callus and sen-callus, relative to mat-callus, in the two Eucalyptus species. We found 15 genes with such regulations in these samples (Table 2), including Eucgr.D00640 (stem-specific protein TSJT1), Eucgr.B00171 (BTB/POZ and TAZ domain-containing protein 1), Eucgr.C00948 (zinc finger CCCH domain-containing protein 20), Eucgr.K01667 (stomatal closure-related actin-binding protein 3), Eucgr.C00663 (glutaredoxin-C10) and Eucgr.C00419 (UPF0481 protein At3g47200). We showed the expression levels of six of these genes in Figure 2E. It is interesting that the expression patterns of all these genes showed an “N” shape during the callus development of both Eucalyptus species. For example, the expression level of Eucgr.D00640 decreased from pri-callus to mat-callus, went up from mat-callus to SRS-callus, and then went down in sen-callus.

**Table 2:** Callus devleopment associated genes identified in the two Eucalyptus species.

GeneID	A4 vs A3			A5 vs A3			B4 vs B3			B5 vs B3			Description
	Log2FC	FDR	Regulation	Log2FC	FDR	Regulation	Log2FC	FDR	Regulation	Log2FC	FDR	Regulation	
Eucgr.C00733	1.32	2.51E-03	UP	-1.42	8.56E-04	DOWN	1.48	4.42E-04	UP	-1.13	1.60E-02	DOWN	uncharacterized LOC104436353
Eucgr.D00640	3.29	1.32E-14	UP	-5.14	3.13E-28	DOWN	2.63	3.08E-10	UP	-4.89	4.07E-26	DOWN	stem-specific protein TSJT1
Eucgr.B00171	1.76	3.08E-05	UP	-3.61	3.72E-16	DOWN	2.01	1.28E-06	UP	-3.43	7.10E-15	DOWN	BTB/POZ and TAZ domain-containing protein 1
Eucgr.C00948	2.25	8.81E-08	UP	-1.88	7.15E-06	DOWN	1.41	8.32E-04	UP	-1.52	5.37E-04	DOWN	zinc finger CCCH domain-containing protein 20
Eucgr.J00388	1.89	7.29E-06	UP	-1.78	2.27E-05	DOWN	1.63	9.11E-05	UP	-2.05	1.71E-06	DOWN	E3 ubiquitin-protein ligase MIEL1
Eucgr.A02118	1.35	1.83E-03	UP	-2.41	8.00E-09	DOWN	1.14	8.06E-03	UP	-1.98	4.10E-06	DOWN	beta-glucosidase BoGH3B
Eucgr.B00946	1.79	2.20E-05	UP	-1.64	9.29E-05	DOWN	2.05	8.96E-07	UP	-1.27	5.71E-03	DOWN	phosphoethanolamine N-methyltransferase 1
Eucgr.K01667	1.56	2.89E-04	UP	-1.26	4.02E-03	DOWN	1.14	8.17E-03	UP	-1.94	9.06E-06	DOWN	stomatal closure-related actin-binding protein 3
Eucgr.I02209	1.79	2.28E-05	UP	-6.17	6.26E-33	DOWN	2.40	9.16E-09	UP	-3.30	6.38E-13	DOWN	monosaccharide-sensing protein 2
Eucgr.J00733	1.03	2.39E-02	UP	-3.43	1.34E-14	DOWN	1.45	7.08E-04	UP	-1.03	3.46E-02	DOWN	ubiquitin-conjugating enzyme E2 20
Eucgr.K01283	5.32	9.76E-30	UP	-1.05	2.65E-02	DOWN	4.70	1.24E-25	UP	-5.31	1.10E-25	DOWN	non-specific lipid-transfer protein 1
Eucgr.F03138	2.84	1.64E-11	UP	-1.13	8.51E-03	DOWN	2.20	1.11E-07	UP	-2.17	4.10E-07	DOWN	beta-fructofuranosidase, insoluble isoenzyme CWINV1
Eucgr.D02029	2.49	3.32E-09	UP	-1.31	2.40E-03	DOWN	3.65	2.44E-17	UP	-1.68	1.98E-04	DOWN	
Eucgr.C00663	-1.62	1.96E-04	DOWN	1.15	7.47E-03	UP	-1.65	9.68E-05	DOWN	1.27	4.82E-03	UP	glutaredoxin-C10
Eucgr.C00419	-1.68	3.47E-04	DOWN	2.07	1.54E-06	UP	-4.03	1.35E-17	DOWN	1.08	2.18E-02	UP	UPF0481 protein At3g47200

Another group of genes involved in the callus development of Eucalyptus include the DEGs during the process from pri-callus to mat-callus to SRS-callus. In *E. camaldulensis*, a Venn diagram of DEGs identified in this process revealed that A4 vs A3 shared 14 upregulated and 146 downregulated genes with A3 vs A2 (Figure 2F, additional file 1). In *E. grandis x urophylla*, we identified 50 upregulated and 145 downregulated genes shared by B3 vs B2 and B4 vs B3 (Figure 2F, additional file 1). Next, we compared the shared DEGs in the two Eucalyptus species during the callus development process. It showed 4 upregulated and 41 downregulated genes shared by the two Eucalyptus species in this process (additional file 1), including Eucgr.B00168 (probable pectate lyase 18), Eucgr.E01615 (putative expansin-B2), Eucgr.K03562 (transcription factor MYB108), Eucgr.H03379 (GEM-like protein 5) and Eucgr.C03297 (ethylene-responsive transcription factor ERF017). Notably, Eucgr.B00168 and Eucgr.E01615 were downregulated in A5 vs A3 and B5 vs B3, respectively (additional file 1); no downregulated genes in the process of pri-callus to SRS-callus were found to be upregulated in sen-callus in both Eucalyptus species; and 17 downregulated genes were also downregulated in the sen-callus in both Eucalyptus species (additional file 1), including Eucgr.K03562, Eucgr.H03379 and Eucgr.C03297.

### Key genes associated with high regeneration ability

We next investigated the genes related to high propagation ability of Eucalyptus. Due to the missing annotation of some genes in Eucalyptus genome, we first examined the expression profiles of previously reported vegetative propagation ability related genes, including ARF19, SERK, LEC and WUS. As shown in additional file 3, two genes (Eucgr.C02178, Eucgr.C03293) were found to encode ARF19 and only Eucgr.C02178 was downregulated between SRS-callus and mat-callus in both Eucalyptus species. We also found two genes (Eucgr.B01219, Eucgr.K03421) encoding LEC14B and none of them were changed in the callus tissues of *E. grandis x urophylla*(additional file 3). Among the three genes (Eucgr.H03383, Eucgr.I01078, Eucgr.F04151) encoding SERK, we found that Eucgr.I01078 was the dominant one. Eucgr.I01078 was not changed in the callus tissues of *E. camaldulensis* and peaked in B3 (additional file 3). It is interesting that Eucgr.F02320 encoding WUSCHEL-related homeobox 4 was upregulated in A3 vs A2, downregulated in B3 vs B2, and upregulated in B4/B5 compared to B3 (additional file 3).

**Fig. 3:**
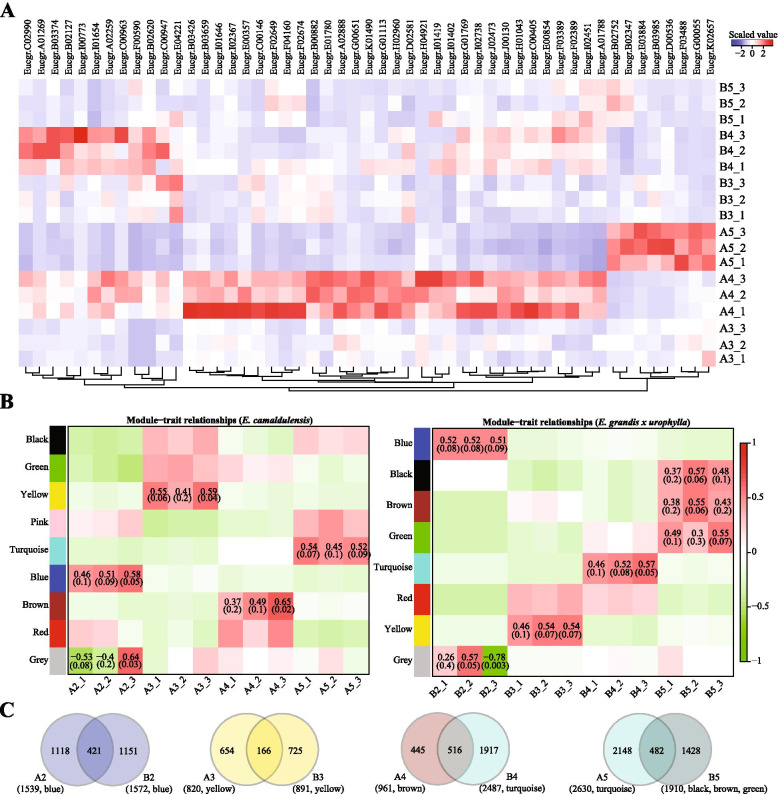
Regeneration ability associated genes and WGCNA. (A) Heat map of high vegetative propagation ability associated genes in the callus samples of the two Eucalyptus species. (B) WGCNA showed the co-expressed genes during the callus development of *E. camaldulensis* (left) and *E. grandis x urophylla* (right). (C) Venn diagrams of shared co-expressed genes at each developmental stages of *E. camaldulensis* and *E. grandis x urophylla*

Then, we analysed the 116 genes upregulated in A4 (compared to A3) and downregulated in A5 (compared to A3), and found 43 genes (Table 3) downregulated or with no changed in B4 (compared to B3), including Eucgr.F00590 (snakin-2), Eucgr.F02674 (putative laccase-9), Eucgr.A02259 (two-component response regulator ARR9), Eucgr.G01769(auxin transporter-like protein 5), Eucgr.B02127 (GRF1-interacting factor 1) and Eucgr.C00947 (transcription factor MYB36). The second group may contain genes downregulated in A4 (compared to A3), upregulated in A5 (compared to A3), but upregulated or not changed in B4 (compared to B3). Using these filters, we identified 8 genes (Table 3), such as Eucgr.B02752 (laccase-7), Eucgr.B03985 (transcription factor MYB108), Eucgr.D00536 (GDSL esterase/lipase At5g45920) and Eucgr.B02347 (scarecrow-like protein 34). A heat map (Figure 3A) showed the expression patterns of these 51 genes in the callus tissues of the two Eucalyptus species. It is interesting that there were 12 genes highly expressed in B3 and B4 but not changed between them, and they were upregulated in A4 compared to A3, such as Eucgr.C02990 (zinc finger CCCH domain-containing protein 2), Eucgr.A01269, Eucgr.B03374 (two-component response regulator ARR6) and Eucgr.B02127 (GRF1-interacting factor 1).

**Table 3:** High propagation ability associated genes in Eucalyptus.

GeneID	A4 vs A3		A5 vs A3		B4 vs B3		B5 vs B3		Description
	log2FC	FDR	log2FC	FDR	log2FC	FDR	log2FC	FDR	
Eucgr.F00590	2.13	3.89E-07	-1.80	1.67E-05	0.09	8.85E-01	-0.57	2.90E-01	snakin-2
Eucgr.F02674	1.84	1.46E-05	-1.50	4.74E-04	-0.93	3.80E-02	-0.04	9.67E-01	putative laccase-9
Eucgr.I01646	1.02	2.50E-02	-2.61	7.28E-10	0.52	2.88E-01	0.54	3.20E-01	ribonucleoside-diphosphate reductase small chain
Eucgr.I01402	1.16	8.59E-03	-1.18	5.71E-03	2.45	4.24E-09	2.08	1.19E-06	subtilisin-like protease SBT1.6
Eucgr.I00773	1.94	3.88E-06	-1.90	6.11E-06	3.99	5.72E-20	0.12	8.73E-01	cytochrome P450 81E8
Eucgr.A02259	2.06	6.33E-06	-2.43	1.07E-05	0.68	1.78E-01	-1.44	5.13E-03	two-component response regulator ARR9
Eucgr.F03389	1.25	5.44E-03	-2.12	1.91E-06	1.71	7.18E-05	1.14	1.75E-02	uncharacterized LOC104450184
Eucgr.A01788	1.02	2.38E-02	-1.73	3.80E-05	0.42	4.01E-01	0.52	3.41E-01	protein STRUBBELIG-RECEPTOR FAMILY 3
Eucgr.J00130	1.47	8.30E-04	-1.22	6.59E-03	0.82	7.80E-02	-0.24	7.20E-01	mini-chromosome maintenance complex-binding protein
Eucgr.G01769	2.22	2.55E-07	-2.83	1.65E-09	0.78	9.25E-02	-0.31	6.38E-01	auxin transporter-like protein 5
Eucgr.A02888	1.19	7.30E-03	-2.40	1.92E-08	0.29	6.03E-01	-1.17	1.46E-02	beta-fructofuranosidase, soluble isoenzyme I
Eucgr.D02581	1.73	5.13E-05	-1.16	8.55E-03	0.70	1.32E-01	-1.47	1.21E-03	protein NUCLEAR FUSION DEFECTIVE 4
Eucgr.F02389	2.02	1.70E-06	-3.03	5.18E-12	0.82	6.87E-02	0.00	1.00E+00	lysine histidine transporter-like 8
Eucgr.C00963	3.36	1.04E-14	-1.26	5.33E-03	3.77	5.62E-18	-2.37	2.25E-07	uncharacterized LOC104436549
Eucgr.I02451	1.23	5.27E-03	-1.67	7.39E-05	0.98	2.63E-02	1.08	2.08E-02	
Eucgr.B03374	2.55	1.52E-09	-2.41	1.56E-08	1.29	2.47E-03	0.62	2.44E-01	two-component response regulator ARR6
Eucgr.G00651	1.20	7.36E-03	-1.98	5.43E-06	0.69	2.25E-01	0.00	1.00E+00	beta-xylosidase/alpha-L-arabinofuranosidase 2
Eucgr.J02473	1.25	6.05E-03	-1.43	1.78E-03	1.89	1.70E-05	0.66	2.54E-01	F-box protein At4g35930
Eucgr.B02127	2.15	3.47E-07	-1.82	1.81E-05	2.52	1.53E-09	-0.99	3.66E-02	GRF1-interacting factor 1
Eucgr.H02960	1.39	1.30E-03	-1.81	2.04E-05	0.16	7.81E-01	-0.88	7.30E-02	acid phosphatase 1
Eucgr.I02738	1.16	9.56E-03	-1.21	5.21E-03	0.63	1.91E-01	0.32	6.04E-01	uncharacterized protein C594.04c
Eucgr.A01269	2.96	1.54E-11	-3.82	6.19E-12	8.78	2.27E-43	0.00	1.00E+00	
Eucgr.B02620	3.95	2.73E-19	-2.09	1.40E-06	0.77	8.82E-02	-0.66	2.00E-01	defensin Ec-AMP-D2
Eucgr.C00947	1.15	1.11E-02	-1.37	1.89E-03	-0.48	3.37E-01	-2.09	1.48E-06	transcription factor MYB36
Eucgr.K01490	1.33	2.57E-03	-1.44	9.35E-04	1.28	3.32E-03	0.04	9.62E-01	short-chain type dehydrogenase/reductase
Eucgr.E00854	1.15	1.16E-02	-2.57	2.36E-08	0.38	4.74E-01	-0.28	6.84E-01	DNA primase small subunit
Eucgr.E04221	1.09	1.59E-02	-3.24	1.99E-13	-1.22	4.18E-03	-1.42	1.34E-03	
Eucgr.H01043	1.54	3.42E-04	-1.84	1.87E-05	0.59	2.16E-01	0.20	7.66E-01	DNA replication licensing factor MCM7
Eucgr.B03659	1.93	6.45E-06	-1.16	9.24E-03	2.09	6.52E-06	0.00	1.00E+00	beta-glucosidase 12
Eucgr.B00882	1.19	7.32E-03	-1.23	4.71E-03	-0.89	4.77E-02	-2.23	4.02E-07	
Eucgr.I01419	1.85	1.31E-05	-1.84	1.98E-05	2.47	5.37E-09	0.23	7.41E-01	probable BOI-related E3 ubiquitin-protein ligase 2
Eucgr.C00146	1.27	3.69E-03	-3.49	7.69E-16	-1.22	4.36E-03	-2.53	3.45E-09	serine carboxypeptidase-like 18
Eucgr.G01113	1.29	3.14E-03	-1.85	9.86E-06	-0.14	8.09E-01	-1.24	6.46E-03	serine carboxypeptidase-like 18
Eucgr.I01654	1.33	2.12E-03	-1.63	1.19E-04	0.98	2.61E-02	-3.55	1.70E-15	chloride channel protein CLC-b
Eucgr.B03426	1.18	7.71E-03	-1.17	6.93E-03	0.86	6.02E-02	0.18	8.00E-01	HVA22-like protein c
Eucgr.I02367	1.30	3.07E-03	-2.08	1.26E-06	0.49	3.32E-01	0.69	1.94E-01	probable LRR receptor-like serine/threonine-protein kinase At4g36180
Eucgr.C00405	1.12	1.28E-02	-2.27	1.66E-07	0.62	1.93E-01	-0.30	6.39E-01	DNA replication licensing factor MCM5
Eucgr.H04921	1.06	2.02E-02	-1.07	1.60E-02	0.09	9.47E-01	0.00	1.00E+00	G-type lectin S-receptor-like serine/threonine-protein kinase LECRK3
Eucgr.F04160	2.30	4.37E-08	-1.51	3.56E-04	-1.02	1.95E-02	-0.15	8.22E-01	putative laccase-9
Eucgr.F02649	2.03	1.36E-06	-1.67	7.46E-05	-0.30	5.73E-01	1.10	1.71E-02	putative laccase-9
Eucgr.E01780	1.95	6.39E-06	-1.08	2.05E-02	-1.21	5.64E-03	-1.64	2.45E-04	non-specific phospholipase C3
Eucgr.C02990	1.56	2.50E-04	-2.20	1.25E-07	1.00	2.21E-02	-0.99	3.55E-02	zinc finger CCCH domain-containing protein 2
Eucgr.E00357	1.97	4.88E-06	-1.11	1.59E-02	-3.72	8.85E-14	-2.38	7.05E-07	expansin-like B1
Eucgr.K02657	-1.51	5.42E-04	1.10	1.11E-02	-0.58	3.15E-01	1.51	2.07E-03	leucoanthocyanidin reductase
Eucgr.E03884	-2.03	1.32E-06	1.62	1.09E-04	-3.69	1.12E-17	-1.80	3.07E-05	uncharacterized LOC104445607
Eucgr.B03985	-2.64	1.13E-09	2.09	6.02E-07	-1.65	9.50E-05	-2.30	1.10E-07	transcription factor MYB108
Eucgr.G00055	-1.06	2.85E-02	1.25	4.14E-03	0.00	1.00E+00	0.00	1.00E+00	
Eucgr.B02752	-2.17	4.55E-07	1.19	5.43E-03	-0.59	2.29E-01	1.78	4.97E-05	laccase-7
Eucgr.F03488	-1.29	4.03E-03	1.15	7.78E-03	-3.35	5.45E-13	-0.84	1.05E-01	protein O-linked-mannose beta-1,4-N-acetylglucosaminyltransferase 2
Eucgr.D00536	-1.15	1.27E-02	1.81	1.80E-05	0.25	6.66E-01	-1.49	1.12E-03	GDSL esterase/lipase At5g45920
Eucgr.B02347	-1.45	1.14E-03	1.12	1.00E-02	-2.89	1.06E-10	0.64	2.37E-01	scarecrow-like protein 34

### WGCNA

We next performed the weighted genes co-expression network analysis to identify co-expressed genes during the callus development process of the two Eucalyptus species. As shown in Figure 3B (left panel), the grey (8 genes) and blue (1,539 genes), yellow (820 genes), brown (961 genes), and turquoise (2,630 genes) modules of genes were identified to be correlated with A2, A3, A4 and A5, respectively. Notably, the yellow module of genes (co-expressed in A3) contained some transcription factor genes, such as Eucgr.C02208 (transcription factor bHLH35), Eucgr.I00291 (ethylene-responsive transcription factor ABR1), Eucgr.K01542 (transcription factor MYB44) and Eucgr.C01943 (probable WRKY transcription factor 40). Next, we showed the co-expressed genes in the callus development process of *E. grandis x urophylla* (Figure 3B, right panel). It is notable that there were 891 genes from the yellow module co-expressed in B3. Then, we compared the co-expressed genes at the same developmental stages of the two Eucalyptus species. It showed that 421, 166, 516 and 482 genes were co-expressed in pri-callus, mat-callus, SRS-callus and sen-callus of the two Eucalyptus species, respectively (Fig. 3C).

### qRT-PCR

**Fig. 4:**
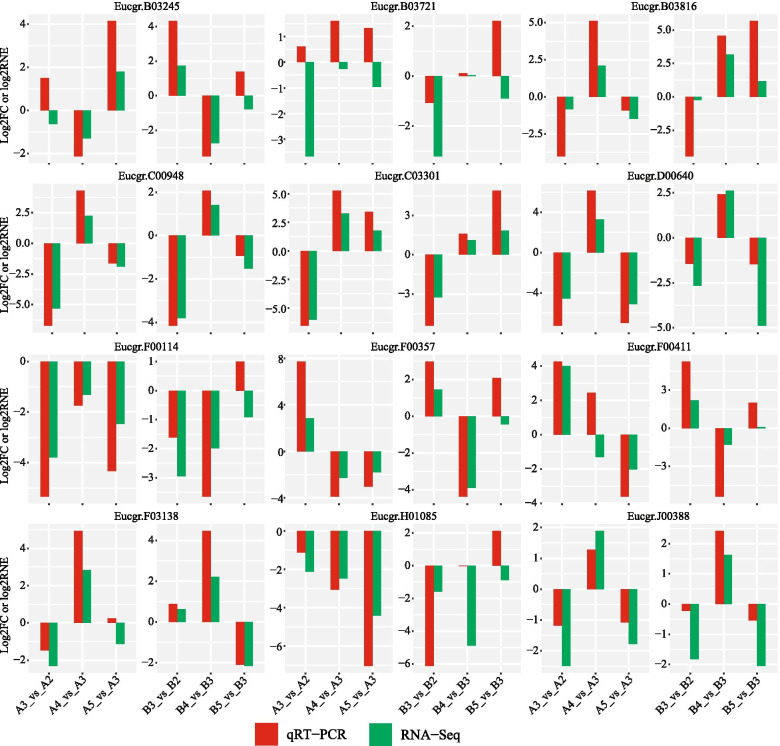
qRT-PCR experiment. A total of 12 genes were selected for qRT-PCR validation and the H2B gene was used as internal control. Overall, 61 (84.72%) out of the 72 events were agreed by both qRT-PCR and RNA-Seq.

We next selected 12 genes and performed qRT-PCR to validate their expression patterns in the callus development of the two Eucalyptus species. The H2B gene was used as the internal control gene. The primer sequences of them can be accessed in additional file 4. We performed three reactions for each gene in one biological sample and a total of 9 reactions were used for one gene (n=9). For the qRT-PCR experiment we used log2RNE values to present the gene changes in the comparisons (mat-callus used as the control), and for transcriptome we used log2FC to show the gene changes. For the comparison of mat-callus and pri-callus (A3 vs A2 and B3 vs B2), we used -log2RNE to show the expression changes. Thus, in total there were 72 events (12 × 3 × 2) that to be validated and we have 61 (84.72%) events were agreed by both qRT-PCR and deep sequencing (Figure 4). It is notable that the dysregulation of some genes was confirmed by both experiments in *E. camaldulensis* and *E. grandis x urophylla*, such as Eucgr.B03816 (transcription factor LHW), Eucgr.C00948 (zinc finger CCCH domain-containing protein 20), Eucgr.C03301 (protein TIFY 10a), Eucgr.D00640 (stem-specific protein TSJT1) and Eucgr.J00388 (E3 ubiquitin-protein ligase MIEL1). High agreement of gene expression patterns in transcriptome sequencing and qRT-PCR indicate that the genes we found in this study might be associated with the callus development and differentiation of Eucalyptus. Their functions require more experiments to be explored.

### Discussion

In this study we analysed the transcriptome profiles during the callus maturation and development processes in two Eucalyptus species with different vegetative propagation capacity. The utilization of successful (SRS-callus) and failed (sen-callus) vegetative propagation callus tissues as contrast strongly support that the genes identified in this study might play important roles in the callus development and might be associated with the vegetative propagation ability in Eucalyptus.

In Eucalyptus, three major stages of regeneration from tissue culture have been reported – co-cultivation, callus induction and shoot regeneration [20]. With our observation, after the dedifferentiation of stem tissue, pri-callus tissues still have low regeneration ability and require further incubation on CIM (Figure 1A and B), which agrees with the callus culture in maize [21]. Not many studies have focused on the callus maturation process in plant and very little is known about the molecular changes in this process. Prior to transfer to the MS medium, fine chopping and partial desiccation of embryogenic calli can simulate the rapid maturation of somatic embryos in date palm [22]. In *Zea mays*, Maturation of somatic embryos was enhanced by transferring the embryogenic callus after 3 weeks to medium containing 6% sucrose and lacking2,4-D [23]. Many genes have been reported to be dysregulated during the embryo maturation in maize, such as histone and ribosomal protein genes, and genes encoding hydrolytic enzymes (nucleases, glucosidases and proteases) and a few storage genes (an α-zein and caleosin) [21]. In this study, we also identified many dysregulated genes during the callus maturation (Figure 2A, additional file 1), including genes encoding histone, ribosomal proteins, nuclease, glucosidases and proteases. Notably, some genes were found to be continually up or down regulated from stem tissue to mature callus (Table 1), such as Eucgr.A02688 (ABC transporter G family member 4), Eucgr.B02604 (G-type lectin S-receptor-like serine/threonine-protein kinase LECRK2), Eucgr.E01615 (putative expansin-B2), Eucgr.A00514 (auxin transporter-like protein 4), Eucgr.C03807 (transcription factor bHLH68) and Eucgr.F01151 (early nodulin-like protein 2). ABCG14, a homologue of ABCG4, has been proved to be a positive regulator of plant growth and play an important role in the major root-to-shoot (acropetal) long-distance cytokinin (CK) transport via the xylem sap [24–27]. Another ABCG4 homologue ABCB4 is an auxin influx transporter which mediates the transport of auxin in roots and contributes to the basipetal transport in hypocotyls and root tips by establishing an auxin uptake sink in the root cap [28, 29]. Eucgr.E01615, which encodes the EXPB2 protein, was upregulated during the callus maturation process (additional file 1). Interestingly, four beta-expansin genes were found to be induced by treatment with gibberellin and by wounding in rice and correlated with rapid elongation of deep-water rice internodes [30]. Further, EXPB2 was found to be a root-predominant gene and play a key role in the root-hair formation in rice [31]. The expression pattern of Eucgr.A00514 (auxin transporter-like protein 4, LAX4) was downregulated in mat-callus compared to pri-callus in the two Eucalyptus species (additional file 1). It is consistent with the discovery of LAX3 gene in rice after grafting [32]. The dysregulation of these genes may suggest that they might play an essential role from the beginning of wounding to encourage healing and preparation for downstream rapid development.

After maturation, shoot regeneration is another important stage indicating the successful propagation. In maize, genes encoding photosynthetic and other chloroplast components (e.g., chlorophyll a/b binding protein) were upregulated as shoots began to green [21]. In the present study, we found that various genes encoding chloroplast components were dysregulated in SRS-callus and mat-callus in the two Eucalyptus species, including 9 genes encoding chlorophyll a-b binding proteins (additional file 1). Interestingly, these 9 genes were downregulated during the callus maturation but upregulated in the shoot regeneration process in Eucalyptus. We also found two genes (Eucgr.H00220 and Eucgr.F03055) encoding photosynthetic NDH subunit of subcomplex B 5, chloroplastic upregulated in B4 vs B3 only (additional file 1). Considering our study used the sen-callus as a contrast of SRS-callus, which may provide a deep insight into the genes associated with the shoot regeneration process of callus, there were 15 genes with diverse regulations in SRS-callus and sen-callus compared to mat-callus (Table 2), such as Eucgr.D00640, Eucgr.B00171, Eucgr.C00948, Eucgr.K01667, Eucgr.C00663 and Eucgr.C00419. Most of these 15 genes have not been reported to be associated with callus development or shoot regeneration. However, some of them have been proved to function in plant development. For example, the protein product of Eucgr.B00171 is BTB/POZ and TAZ domain-containing protein 1, which is a substrate-specific adapter of an E3 ubiquitin-protein ligase complex and involved in gametophyte development [33]. Eucgr.C00419 encodes the UPF0481 protein At3g47200, which has been reported to be upregulated during the early flower development in *Prunus mume* [34]. Eucgr.D00640 encodes the stem-specific protein TSJT1, which has been found in other plants like tobacco, grape, and soybean. The functions of these newly callus development associated genes require more experiments to investigate their roles in somatic embryogenesis, dedifferentiation, differentiation, and development.

Known vegetative propagation ability associated genes (e.g., ARF19, SERK, LEC and WUS) have been reported to play key roles during the dedifferentiation process in Eucalyptus [19], however, we did not find strong association between these genes and the callus development as we cannot determine their expression patterns in this process (additional file 3). However, because the two Eucalyptus species used in this study have distinct ability of vegetative propagation, we identified 51 genes that might be related to their somatic embryogenesis potential (Table 3) and 29 of them have been reported in the dedifferentiation process of Eucalyptus [19], including Eucgr.F02674 (putative laccase-9), Eucgr.B03374 (two-component response regulator ARR6), Eucgr.C00947 (transcription factor MYB36). These 29 genes might be triggered during the early dedifferentiation process and the other 22 genes might be induced at the callus maturation and shoot regeneration processes, such as Eucgr.A01788 (SRF3, protein STRUBBELIG-RECEPTOR FAMILY 3) and Eucgr.B02127 (GIF1, GRF1-interacting factor 1). SRF3 has been proved to be involved in the plant immunity [35]. Recently, it was showed to be associated with the cell proliferation during the switch development from the apical buds to leaf marginal tissues [36]. Interestingly, its homologue SRF4 was shown to play an important role in making plants display enlarged leaves through affecting cell wall formation [37]. As a transcription coactivator, GIF1 has also been shown to control cell proliferation. Being a target of miR396, GIF1 and other GIFs act in the regulation of meristem function, at least partially through the control of cell proliferation [38]. In addition, together with GRF5 GIF1 controls the development of appropriate leaf size and shape through the promotion and/or maintenance of cell proliferation activity in leaf primordia, GIF1 plays a role in adaxial/abaxial patterning and growth in leaf morphogenesis, and together with GATA18/HAN, GIF1 mediates the cotyledon identity by preventing ectopic root formation through the repression of PLT1 expression [39]. We assume that the dysregulation of the 22 genes might be regulated by some mechanisms and the interaction network requires more experiments to be explored. When and how the vegetative propagation ability associated genes are expressed during the callus development are also valued research areas and will be focused in the future.

## Conclusions

In conclusion, we analyzed the transcriptome profiles of callus tissues during the maturation and shoot regeneration processes of two Eucalyptus species which have distinct vegetative propagation ability. We observed that the regeneration rates of the tissue culture induced callus by stem peaked at 21 days of incubation on CIM. In the callus maturation process we identified 3,790 and 3,740 DEGs in *E. camaldulensis* and *E. grandis x urophylla*, respectively, including genes encoding histone/ribosomal proteins and genes involved in the plant hormone signalling transduction pathway. Then, using SRS-callus and sen-callus as contrast we identified 15 genes (e.g., Eucgr.D00640, Eucgr.B00171, Eucgr.C00948, Eucgr.K01667, Eucgr.C00663) which might play important roles during the development of mat-callus. They were annotated to encode the stem-specific protein TSJT1, zinc finger proteins, stomatal closure-related actin-binding proteins and glutaredoxin-C10 proteins. Further, 51 genes were identified to be associated with the ability of somatic embryogenesis of Eucalyptus, of which 22 genes (e.g., SRF3 and GIF1) might be induced after the dedifferentiation. This is the first time to study the transcriptome profiles of callus development in Eucalyptus. The results will improve our understanding of gene regulations and molecular mechanisms in the callus development and vegetative propagation of Eucalyptus. More importantly, the output of this study may benefit the Eucalyptus breeding program.

## Methods

### Plant material and culture conditions

The original seeds of *E. camaldulensis* (voucher ID: c0009) and *E. grandis x urophylla* (voucher ID: j0017) were obtained from the wild in 1984 without any restrictions. Then, the seeds and plants were confirmed by a senior botanist Prof. Dongyun Xiang and they were maintained in the experimental fields of Guangxi Forestry Research Institute. The stem tissues were obtained from the *in vitro* tissue-culture induced seedlings of *E. camaldulensis* (voucher ID: c0009, A1) and *E. grandis x urophylla* (B1) trees, and maintained on the callus induction MS medium (CIM, supplemented with 20mg/L Ca(NO_3_)_2_, 1 mg/L KT and 0.5 mg/L 2,4-D) for 10 days to get the pri-callus (A2, B2). Then, the pri-callus was continually incubated on the CIM for another 11 days to get the mature callus (A3, B3), which was transferred onto the shooting-inducing medium (SIM, MS medium supplemented with 20 mg/L Ca (NO_3_)_2_ +2.0 mg/L 6-BA + 0.2 mg/L NAA) for incubation. The callus was incubated on the SIM for 10 days to get the shoot regeneration stage callus (A4, B4), which were developed with some buds as successful propagation. While the mature callus was incubated on the CIM for another 14 days, we get the senescence callus (A5, B5) which totally lose the embryogenic capacity.

### Total RNA extraction, cDNA library preparation and transcriptome sequencing

Total RNA was extracted from the plant tissues (A2~A5, B2~B5) using the TRIzol reagent, as previously described [19, 40]. Then, Agilent 2100 Bioanalyzer was used to evaluate the quantity and quality of the total RNA samples. Equal amount of total RNA (1 μg) was used for the cDNA library construction, as described [19]. In brief, the poly-A mRNAs were enriched using the magnetic oligo (dT) beads and then were fragmented into 200 bp pieces. Next, random hexamer (N6) primers were used to build double strand cDNA libraries for all the samples. After the libraries were end-repaired by using phosphate at the 5’ end and sticky ‘A’ at the 3’ end, they were ligated with sequencing primers for BGISEQ-500. The libraries were sequenced on the BGISEQ-500 RS platform with paired-end 150 strategy.

### Genome alignment, gene expression profiles and differential expression analysis

Raw reads were processed by SOAPnuke to remove sequencing adaptors, low quality reads and contamination reads [41]. Then, the clean reads were aligned to the Eucalyptus genome (v2.0, https://plantgenie.org) using hisat2 [42]. Stringtie [42] and Subread [43] were used to profile gene expression for each sample and count the read counts aligned to each gene, respectively. We next used the TPM (transcripts per million reads mapped) method to normalize gene expression in each sample and filtered lowly expressed genes (average TPM < 5). To perform differential expression analysis, we first calculated the coefficient of variation (CV) of each gene and genes with CV > 0.5 were filtered. Then, we employed edgeR with some cut-offs, including log2 fold change (log2FC) > 1 or < -1, p-value < 0.05, false discovery rate (FDR) < 0.1, to identify differentially expressed genes in two samples.

### Functional analysis

We next annotated the Eucalyptus genes by mapping them to the Gene Ontology (GO) and KEGG pathway databases, as previously described [44]. Then, the enriched GO terms and KEGG pathways by differentially expressed genes were identified by p-value (< 0.05), calculated by Fisher’s exact test, and q-value (< 0.05), calculated by the R package ‘qvalue’.

### WGCNA

We used the R package “WGCNA” to identify co-expressed genes during the callus development of the two Eucalyptus species [45], according to the manufacturer’s protocol.

### RT-PCR

We selected 12 genes for qPCR-PCR validation and used the H2B gene as the internal control. The primers of these 13 genes were predicted by Primer3 and synthesized at BGI-Shenzhen. The procedure of qRT-PCR was same as a previous study [19, 46]. For each gene in a biological replicate, we performed three qRT-PCR reactions and in total we have 9 replicates for one gene (n=9). After the Ct values were calculated and averaged, we used the ΔCt value to present the gene expression in each sample. Then, using the mature callus as the control sample we calculated the ΔΔCt value to show the difference of a gene in the callus development. Last, relative normalized expression (RNE) was used to show the gene expression change: *RNE* = 2^−ΔΔCt^ and log2RNE was used to match the transcriptome sequencing method. For pri-callus and mature callus comparison (A3 vs A2 and B3 vs B2), we used -log2RNE to present the gene changes.

## Supplementary Information

**Additional file 1.**

**Additional file 2.**

**Additional file 3.**

**Additional file 4.**

## Data Availability

The raw sequencing data can be accessed from the NCBI Sequence Read Archive (SRA) platform (http://trace.ncbi.nlm.nih.gov/Traces/sra/) under the accession number PRJNA761197.
